# How mites influence cage-free egg production in the United States, mite management strategies, and the mitigating role of genomic selection

**DOI:** 10.1093/af/vfae023

**Published:** 2024-10-14

**Authors:** Jacqueline A Holquinn, Hayley L Sutherland, Elaina R Sculley, Marisa A Erasmus, Luiz F Brito, Amy C Murillo

**Affiliations:** Department of Entomology, University of California, Riverside, CA, USA; Department of Animal Sciences, Purdue University, West Lafayette, IN, USA; Department of Animal Sciences, Purdue University, West Lafayette, IN, USA; Department of Animal Sciences, Purdue University, West Lafayette, IN, USA; Department of Animal Sciences, Purdue University, West Lafayette, IN, USA; Department of Entomology, University of California, Riverside, CA, USA

**Keywords:** breeding program, ectoparasite, layers, novel traits, poultry, welfare

**Implications**

- The US poultry industry is significantly increasing cage free egg production, which is providing opportunities for ectoparasitic mites.
- Ectoparasites negatively impact hen welfare, including hen health, behavior, and productivity, and mite management strategies are lacking.
- There is evidence of genetic variability for mite resistance and genomic selection could offer a feasible alternative for improving mite resistance in purebred and crossbred poultry populations.

## Introduction

Egg production in the United States has changed significantly in the last century. From the 1920s to the 1960s, egg production underwent a significant evolution, driven by advancements in breeding and husbandry practices (Kidd and Anderson, 2019). In the early 20th century, chickens were typically raised on small farms, where they were valued for both egg and meat production. However, during this period, there was a shift toward industrialization of poultry farming (Kidd and Anderson, 2019). Innovations in housing, feeding, and disease management contributed to improved efficiency and productivity within the egg industry. By the 1960s, the adoption of intensive farming methods, including the use of conventional or “battery” cages, had become widespread, dramatically increasing the scale of egg production, and setting the stage for modern commercial poultry farming practices (Kidd and Anderson, 2019). However, a growing sector (approximately 40%) of the egg industry is adopting cage-free housing for over 124 million laying hens as consumer demand for improved animal welfare increases and some state legislation requires more space per bird. There has also been an increase in certified organic egg production, which by definition is cage free; there are over 27.5 million organic laying hens raised in the US annually (USDA-NASS, 2021). The switch from cage-to-cage-free production provides hens with more behavioral opportunities, but some risks to hen health are more prevalent in cage free systems (Lay et al., 2011). In particular, the switch to cage-free housing is significantly impacting the prevalence of ectoparasites such as the northern fowl mite (*Ornithonyssus sylviarum*) and poultry red mite (*Dermanyssus gallinae*) in poultry facilities (Murillo and Mullens, 2016; Chambless et al., 2022). Mite infestations impact all areas of hen welfare, including hen health, behavior, productivity, and emotional state. Here, we discuss what is known about northern fowl mites and poultry red mites, their impact on hens; current mite management strategies and challenges, and the potential for using genomic tools to manage mites in US cage-free egg production.

## Biology of the NFM and Poultry Red Mite

**Figure 1.**
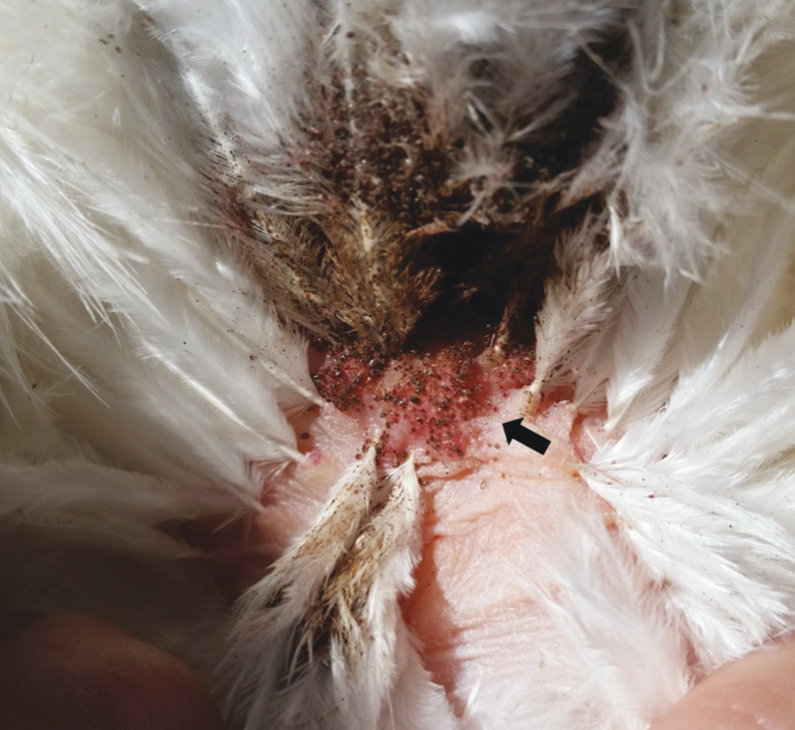
Northern fowl mites live on-host in feathers of the vent region. Adult and protonymph life stages travel to the skin surface to blood feed (black arrow). Photo by A. Murillo.

Currently, the northern fowl mite (NFM) is the most prevalent and damaging ectoparasite of commercial US poultry (Murillo and Mullens, 2017). Northern fowl mites spend their entire life cycle on the feathers of the vent region of their chicken host and can survive for up to 5 wk off-host under ideal conditions (Fig. 1; Chen and Mullens, 2008). The 5 life stages of NFM include the egg, larva, protonymph, deutonymph, and adult stages; NFM must feed on host blood in the protonymph and adult life stages (Sikes and Chamberlain, 1954). It can take 5 to 7 d for NFM to complete their life cycle, which allows populations to grow very quickly (Sikes and Chamberlain, 1954). An unmated female NFM can lay unfertilized eggs which will hatch into males (McCulloch and Owen, 2012). Female mites can then mate with these male offspring to produce fertilized eggs that will hatch into females (McCulloch and Owen, 2012). This reproductive strategy allows for mite populations to be founded by a single virgin female and to grow very quickly.

**Figure 2.**
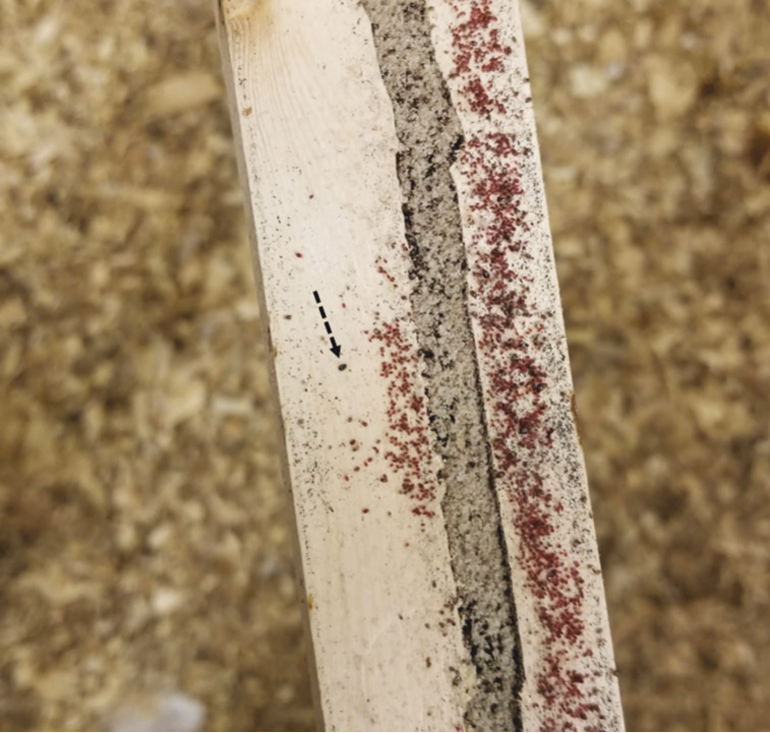
All life stages of the poultry red mite live off-host in the environment. Various poultry red mite life stages are shown on a perch, with an adult mite highlighted (dashed arrow). Photo by A. Murillo.

In other parts of the world, the poultry red mite (PRM) is the most prevalent ectoparasite of laying hens (Sparagano et al., 2014). Poultry red mites also require a blood meal to develop and reproduce, but instead of living on-host, PRM live in cracks and crevices in the environment and travel to their host at night to blood feed (Fig. 2; Axtell, 1999). The PRM can survive up to 34 wk under field conditions without a blood meal (Axtell, 1999). Like NFM, PRM also have 5 life stages, with the protonymph, deutonymph, and adult life stages requiring a blood meal. Development into adults occurs within 8 to 14 d, with populations growing more quickly in summer months (Axtell, 1999; Maurer and Baumgärtner, 1992). Both NFM and PRM can cause anemia or even death when infestation levels are high (reviewed by Murillo and Mullens, 2017; Sparagano et al., 2014, respectively). In the United States, PRM have not been a pest of commercial layers since the widespread adoption of battery cages (Lay et al., 2011). However, recently PRM have been found in cage-free chicken flocks in the United States (Murillo and Mullens, 2016; Chambless et al., 2022). Cage-free poultry housing systems have complex environments that offer harborage for PRM, and this is now a reemerging pest of poultry in the United States (Axtell, 1999; Murillo and Mullens, 2020).

## Effects of NFM and PRM Infestation on the Hen Host

### Immune response

The hen immune response to NFM is induced by mite feeding, and mite-naïve hens will harbor higher mite populations relative to hens reinfested with NFM (DeVaney and Ziprin, 1980). The hens’ immune response involves the creation of targeted antibodies against NFM proteins, including IgY, IgM, an NFM-specific antibody (Minnifield et al., 1993), and skin inflammation (Owen et al. 2009). Previous research demonstrated that blood serum immunoglobulin levels of IgY and IgM were higher for birds continuously infested with PRM (Harrington et al., 2010). Further, elevated IgY levels have been found in eggs produced from infested hens. Akbayev et al. (2020) reported that increased eosinophils and aminotransferase (ALT) were the only 2 measured parameters that were influenced by infestation level. When NFM contact the mite-specific antibodies in the hen’s blood, the antibodies can cause internal damage and further impact mite survivability. However, Minnifield et al. (1993) stated that the presence of antibodies in the blood alone was not enough to affect mites upon ingestion and a stronger antibody response is needed to effectively inhibit NFM blood feeding. As another aspect of hens’ immune response, skin inflammation inhibits NFM blood feeding, resulting in decreased survivability and development first for the protonymphs, then for the adult mites as the skin thickens, preventing mouthparts from reaching capillaries (Owen et al., 2009). Specifically, epithelial proliferation, resulting from migration of immune cells into the skin, helps to form a barrier between capillaries and the outer layer of the dermis (Owen et al., 2009). Despite its potential, no studies to date have targeted this response for vaccine development. Hens infested with NFM have also been shown to have a higher prevalence and greater severity of skin lesions (Murillo et al., 2020), likely associated with this host immune response. There is also a host immune response to PRM antigens; however, the response is not strong enough to influence the mite population (reviewed by Decru et al., 2020).

### Feather condition

Changes in host feather condition may impact the ability of NFM and PRM to effectively infest hens. Jarrett et al. (2022) evaluated different welfare indices in NFM-infested laying hens, and their results indicated that conspecific feather pecking in the vent area resulted in the loss of downy feathers, subsequently leading to decreases in on-host NFM numbers. Feather condition was overall worse for hens infested with NFM compared to control birds, with specific differences found for feather condition of the head, neck, crop, keel, back, rump, and belly (Jarrett et al., 2022). While NFM prefer the down feathers in the vent which provides an ideal microclimate, high NFM loads often result in mites migrating to other locations on the bird, like the tail or the breast (Murillo and Mullens, 2017). NFM infestations cause vent feathers to appear “dirty” from the buildup of mite eggs, cast skins, and feces (Fig. 1; Murillo and Mullens, 2017). Although PRM infestations do not soil feathers like NFM, PRM infestations are associated with changes in feather condition. Few studies regarding PRM effects on feather condition are available, but Schreiter et al. (2022) reported that condition of the feathers worsened as PRM infestation level increased. Skin lesions only tended to be influenced by infestation level, suggesting that lesions might be more related to feather pecking (Schreiter et al., 2022). Poor feather condition associated with mite infestation may reduce hens’ ability to thermoregulate, though this has not been studied.

### Behavior

Preening and dustbathing behavior are the hens’ main mechanisms of maintaining feather health and condition, and irritation from NFM infestation and blood feeding, in addition to the presence of the parasites themselves, could result in increases in hen grooming behavior (Jarrett et al., 2022) in addition to the presence of the parasites themselves. Indeed, Murillo et al. (2020) reported increased preening behavior when hens had high NFM infestation levels, alongside inconsistent increases in dustbathing behavior. Vezzoli et al. (2015) documented that the amount of time spent preening in the vent and uropygial areas increased at peak infestation (6 wk) compared to pre-infestation levels. When observing nocturnal behaviors of white laying hens, hens with higher NFM loads were found to rest less and preen more when compared to hens with fewer mites (Jacobs et al., 2019). Even with low NFM infestation levels, hens still preened more frequently and for an extended period compared to hens without mites (Jacobs et al., 2019). The increased preening may be related to hen discomfort or “itchiness” resulting from ectoparasite infestation (Jacobs et al., 2019), but this has been difficult to quantify in an objective manner.

Poultry red mite infestation similarly increases grooming, dustbathing, and scratching behavior, both in the daytime and nighttime (Kilpinen et al., 2005), and treatment of PRM (eradication) significantly reduces nighttime activity of hens (Temple et al., 2020). In addition to increased preening, PRM infestation is associated with increased feather pecking (Kilpinen et al., 2005) and cannibalistic behavior (reviewed by Sigognault Flochlay et al., 2017).

### Productivity

Several studies have documented decreases in productivity as a result of NFM infestation. As early as 1979, researchers reported 5-15% decreases in egg production and depressed initial body weight of NFM-infested hens compared to control hens as hens reached peak production (DeVaney, 1979). More recently, Jarrett and colleagues (2022) found that NFM infestation negatively affected hen-day egg production (no. eggs produced by the total number of hens present on a given day) and body weight, alongside other measures such as mortality, feather coverage, and shell thickness of brown hens. Hen-day egg production has been found to decrease at least 2% due to NFM infestation, with some research finding up to a 5.5% decrease (Jarrett et al., 2022; Mullens et al., 2009). Poultry red mite infestation level has a similar, positive correlation with productivity loss (Decru et al., 2020), and Kilpinen et al. (2005) reported lowered weight gain for hens infested with PRM compared to controls. However, treatment to reduce PRM levels can lead to a recovery of hen body weight and increased feed intake (Decru et al., 2020). Hens can also become anemic, contributing to increased feed consumption, poor feed conversion, and decreased egg production and quality (Kilpinen et al., 2005). Egg quality is affected such that blood spots form on the shells, making them undesirable (Decru et al., 2020).

## Strategies for Managing NFM and PRM

Integrated pest management (IPM) is an approach for managing pests that combines a variety of tactics to minimize economic damage; more comprehensive IPM approaches for NFM and PRM are reviewed by Axtell (1999) and Decru et al. (2020), respectively. Here, we discuss a variety of different strategies for poultry mite management.

### Pesticides

Historically, the management of NFM has relied heavily on synthetic pesticide use. However, growing concerns from consumers about the potential environmental consequences and lingering effects on poultry products have prompted a reevaluation of traditional approaches. In addition, birds cannot easily be treated with pesticides in cage-free production systems (Murillo and Mullens, 2020). Pesticides used to control NFM need to be sprayed on birds directed toward the vent region at high pressure to ensure the spray penetrates the feathers (Axtell, 1999; Murillo and Mullens, 2017). High-pressure chemical sprays have been applied in conventional cage systems, but this approach is less effective in cage free systems where it is more difficult to spray chemicals on the birds (Murillo and Mullens, 2017). Controlling PRM has similar difficulties as controlling NFM: there are limited pesticides available and there is growing mite resistance to the chemicals that are being used (Marangi et al., 2009; Murillo and Mullens, 2017). In contrast to NFM, PRM live in the environment, requiring pesticides to be applied to structures, focusing on potential areas of harborage (Marangi et al., 2009; Decru et al., 2020). Another method for managing PRM is the addition of fluralaner to drinking water, which has been shown to reduce mite numbers by up to 100% in treated birds (Decru et al., 2020), though this product is not currently available in the US. Alternatives to synthetic pesticides, including plant-derived products and inert dusts, can also be used for mite control, though their efficacy is variable (Decru et al., 2020).

### Biosecurity

Biosecurity remains the most effective method for preventing the introduction and transfer of ectoparasites (Axtell, 1999). Preventing wild birds and rodents from entering poultry facilities reduces the spread of NFM and PRM (Axtell, 1999). Regulating who has access to poultry farms, requiring employees to wear personal protective equipment (PPE), and disinfecting footwear and equipment also limits the spread of ectoparasites (Axtell, 1999). Cleaning, disinfecting, and pesticide application (where possible) are encouraged between flocks to remove ectoparasites that are left in the environment (Axtell, 1999).

### Monitoring

In addition to implementing strict biosecurity practices, monitoring for NFM and PRM is a valuable tool for preventing economic impacts to producers, prompting necessary control before damage occurs (Murillo and Mullens, 2020). Northern fowl mites can be monitored by performing mite counts on designated sentinel hens in a poultry house or keeping track of eggs with mites; 4/100 eggs with mites indicates that 25% of a flock is infested (Murillo and Mullens, 2020). Observing red spots on eggs can be used for PRM monitoring (Decru et al, 2020; Murillo and Mullens, 2020). Corrugated cardboard traps can be placed in poultry houses, usually near nest boxes or perches, to provide an estimate of infestation levels in houses (Decru et al., 2020; Murillo and Mullens, 2020).

### Host factors

*Beak morphology*. It is standard commercial practice to beak-trim laying hens at hatch to prevent issues with aggressive pecking and cannibalism later in life (Mullens et al., 2010). However, trimming alters the morphology of the beak and hinders hens’ ability to combat ectoparasite infestation through preening. Beak-trimmed hens have higher mite scores compared to hens with intact beaks, suggesting that beak trimming renders hens less effective at removing ectoparasites with their beak (Mullens et al., 2010; Vezzoli et al., 2015). When hens are left with intact beaks, NFM infestation levels are low, but cannibalism among hens can become an issue (Jarrett et al., 2022). Docile breeds that do not require beak trimming, and/or different trimming techniques may help birds manage ectoparasite populations more effectively.

### Host factors

*Vaccines*: Vaccines against mites offer an attractive alternative to pesticides and would be allowed for use in organic production in the United States. Several studies have contributed to the development of vaccines against PRM, though none are yet commercially available. Recombinant vaccines were able to significantly reduce PRM population in the environment and increase mite death (Bartley et al., 2015), and a prototype vaccine, which targets hemoglobin digestion in PRM, produced a lasting IgY levels in vaccinated hens, also reducing the number of eggs produced by PRM by ca. 50% (Price et al., 2019). Recently, cysteine proteases were identified that may offer a vaccine solution with cross protection for *Ornithonyssus* spp. and *Dermanyssus* mites (Win et al., 2023).

*Genetic and genomic applications*: Considering the challenges of eliminating mites from poultry farms, there is a growing interest in implementing genetic and genomic technologies for breeding poultry that are more resilient and resistant to NFM infestation in cage free settings. The success of a breeding program relies on the identification of traits that are heritable, repeatable, and capture the biological mechanisms of interest (e.g., laying hen response to NFM or PRM infestation). Recent studies have demonstrated a strong correlation between the hen’s reaction to NFM infestation and changes in behavior, welfare, and inflammatory responses (Owen et al., 2009; Murillo et al., 2016; Murillo et al., 2020). There is also evidence of genetic variability in laying hens’ responses to mite infestation. For instance, previous studies have identified specific genes within white leghorns, such as those linked to the MHC-B-haplotypes, as being correlated with varying degrees of mite resistance (Owen et al., 2009; Murillo et al., 2016). Interestingly, differences in skin inflammation patterns were observed between birds with the mite-resistant B21 haplotype and those with the mite-susceptible B15 haplotype, suggesting that potential variations in immune responses to NFM infestation are based on genetic background (Owen et al., 2009; Murillo et al., 2016). The known genetic variability in traits linked to hen characteristics, NFM sensitivity, immunological response, and the major histocompatibility complex (Lukasch et al., 2017; Chu et al., 2023) suggest that genomic selection might be an effective tool for improving hen resistance and resilience to mite infestation (Table 1). Although there is a lack of studies evaluating the feasibility of breeding for improved resistance to ectoparasites in poultry, there are reports of substantial genetic variability and accurate genomic prediction of breeding values for ectoparasite resistance such as for ticks in cattle (Cardoso et al., 2021).

**Table 1.** Strategies for managing mites associated with egg production (NFM, northern fowl mites; PRM, poultry red mites)

Strategies	Pros	Cons
Pesticides	Specifically target where the mites live (NFM, on-host; PRM, housing environment)	Especially hard to treat birds directly in cage free housing; mites may develop resistance
Biosecurity	May prevent infestation altogether if carefully implemented	Needs to be combined with other strategies for mite elimination; requires personnel cooperation
Flock monitoring	Can identify infestation early on and action can be taken to minimize economic losses	Time consuming and requires training
Beak morphology	Laying hens with untrimmed beaks can preen more effectively to remove mites	Untrimmed beaks can lead to cannibalism issues in commercial flocks
Genetic and genomic applications	Mite resilient poultry strains can be used in any housing system, no additional cost to farmer	Lack of research for implementation in poultry

## Knowledge Gaps and Future Directions

**Figure 3.**
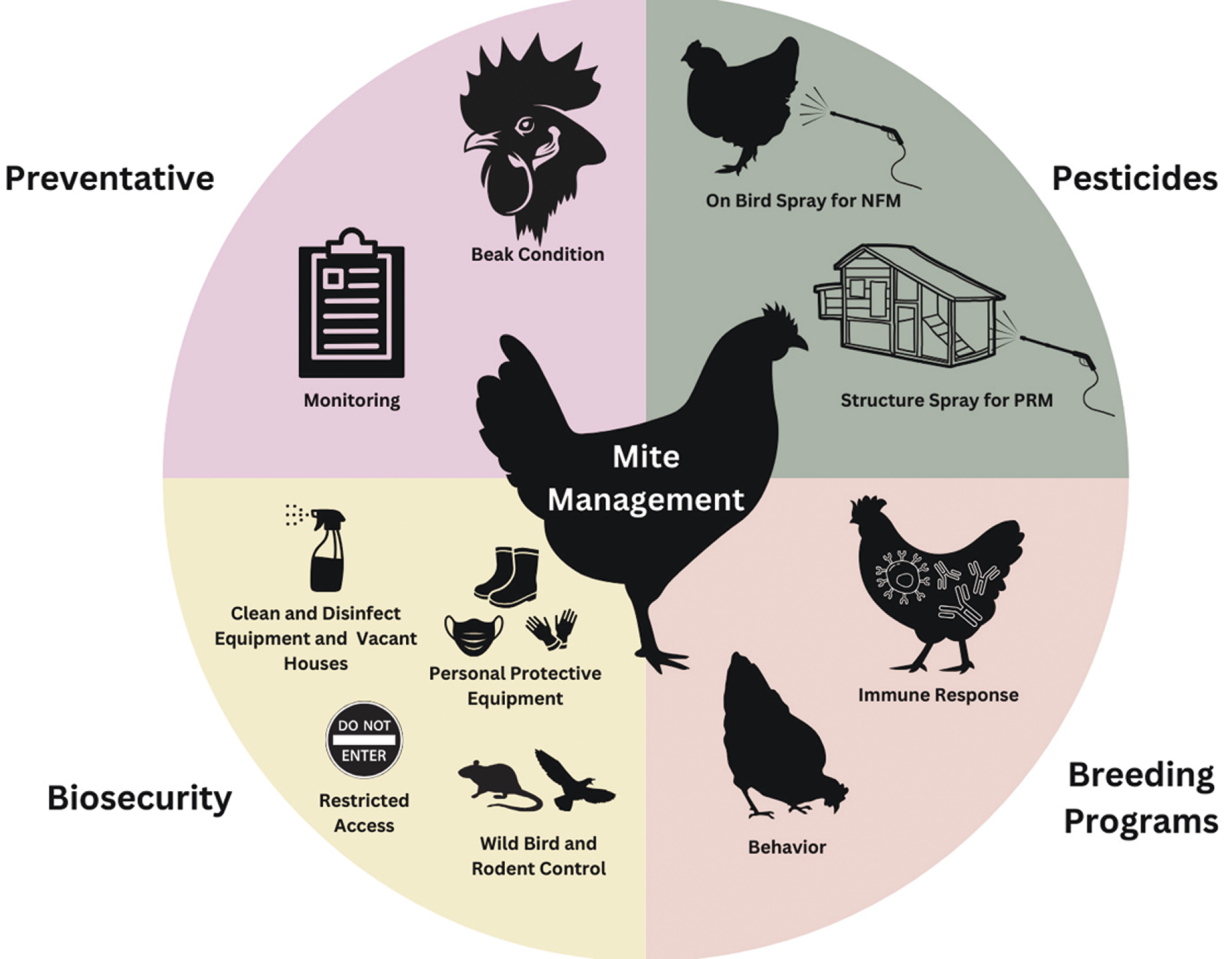
An integrated pest management (IPM) approach to northern fowl mite (NFM) and poultry red mite (PRM) on-farm management will include strategies such as mite prevention, biosecurity, pesticide use, and breeding programs. Figure by J. Holquinn.

This overview of NFM and PRM provides some information about the damaging impact that both pests have on the health, behavior, welfare, and productivity of laying hens, indicating that infestations have severe consequences for hens and egg production. As global demand for animal protein continues to increase alongside changes in global climate, ectoparasites are becoming increasingly problematic (Murillo and Mullens, 2016; Chambless et al., 2022) and more information about the interactions among the parasites and their hosts is needed to identify effective, sustainable strategies for preventing and reducing the detrimental consequences of mite infestation. Given recent changes in layer housing and reemerging threat of mite infestation, there is an urgent need for novel and innovative management strategies to mitigate ectoparasite infestations and enhance overall hen welfare in the evolving landscape of egg production. Control of NFM and PRM, along with other important ectoparasites, needs to adjust with the changing poultry environment, integrating diverse strategies to reduce negative impacts on animal welfare and sustainability (Fig. 3; Decru et al., 2020; Murillo and Mullens, 2017, 2020). With the limitations of pesticide use, one promising strategy for combating mite infestations is to focus on the hens and their ability to resist infestation. Genomic strategies are particularly promising because these strategies can be implemented by breeding companies, thereby influencing the welfare of millions of animals. Considering the structure of modern poultry breeding schemes in which nucleus animals are raised on high-level biosecurity farms, there is a need to develop reference populations (animals with both genomic data and phenotypic records) based on individuals that are genetically related to the selection candidates, as well as a need to evaluate the potential impact of genotype-by-environment interactions on hens’ ability to combat mites. There is also a need to evaluate the genetic relationship of the derived traits. If successful, genomic selection for greater resilience and resistance to NFM and PRM could be an effective approach for improving laying hens’ general health and productivity while reducing the need for chemical intervention. There are several benefits of using this approach as part of a mite management program, including implementation regardless of housing or management type (i.e., organic or conventional). However, genomic strategies take years to develop and refine, and there is a critical need for research to guide the ongoing changes in egg production.

## About the Authors

 **Jacqueline A. Holquinn, MS**: After serving 6 yr in the US Navy, Jacqueline earned a BS in Cell, Molecular, and Developmental Biology from UC Riverside. She completed an MS in Entomology at UC Riverside studying kleptoparasitic mites of solitary bees. Jacqueline is a PhD student in the department of entomology at UC Riverside where she is currently studying ectoparasites of poultry, specifically northern fowl mites, poultry red mites, and bed bugs. She is interested in studying the negative impact that poultry ectoparasites have on their chicken hosts and using cultural control tactics to minimize their impact.

 **Hayley L. Sutherland, MS** is a PhD student in the Department of Animal Sciences at Purdue University. She previously earned a BS in Animal Sciences with a concentration on behavior and well-being from Purdue University. She also received her MS in Animal Sciences with a focus on behavior and welfare studying feed additive effects on broiler chickens. Her current research aims to better understand how ectoparasites affect laying hen behavior and welfare.

 **Elaina R. Sculley** is a PhD student in the Department of Animal Sciences at Purdue University. She holds a Bachelor of Science in Animal Sciences from Colorado State University and a Master of Science in Animal Sciences from the University of Missouri. She also received a certificate in Animal Nutrition from Colorado State University and is working toward earning a certificate in Livestock Data Analysis from Purdue University. Currently pursuing her PhD at Purdue, she focuses on quantitative genomics and animal breeding in livestock, with a particular interest in bioinformatic analyses. Her expertise and research interests lie in understanding and improving livestock genetics for enhanced productivity and welfare.

 **Marisa A. Erasmus** is an associate professor of Animal Behavior and Welfare in the Department of Animal Sciences at Purdue University. She earned her BS and MS degrees from the University of Guelph in Canada and her PhD from Michigan State University. Her research focuses on developing animal-based measures of welfare and examining the effects of environmental and management factors on animal behavior and welfare. She has a strong interest in interdisciplinary research that can help inform and guide management and welfare practices for farm animals. Her extension activities include working with the commercial poultry and livestock industries to understand and address animal welfare needs and providing educational resources about animal production and welfare.

 **Luiz F. Brito** is an associate professor of Quantitative Genetics and Genomics in the Department of Animal Sciences at Purdue University. He earned his BS degree in animal science and MS degree in Genetics and Animal Breeding from the Federal University of Vicosa in Brazil and his PhD in Quantitative Genomics from the University of Guelph in Canada, followed by a 2-yr postdoctoral training at the same university. Since joining Purdue University in 2018, his research program has focused on (1) the integration of multiple data sources (large-scale and high-throughput phenotypes and omics-derived datasets) to reveal the genetic basis underlying the phenotypic variability in livestock behavior, welfare, and climatic resilience; and (2) the development of selection methods and approaches to enable efficient incorporation of these traits into livestock breeding programs.

 **Amy C. Murillo** is an assistant professor of Veterinary Entomology in the Department of Entomology at the University of California Riverside. She earned her BS from Purdue University, MS from North Carolina State University, and PhD from UC Riverside, all in Entomology. Research in her lab aims to better understand how mites, lice, flies, and other pest arthropods affect the behavior, welfare, and production of poultry and other livestock. This includes developing control methods for mitigating economic damage and improving animal welfare and production. **Corresponding author:**amy.murillo@ucr.edu
